# Supplementary material for: Distributed functions of prefrontal and parietal cortices during sequential categorical decisions
Source: eLife. 2021 Sep 7;10:e58782. doi: 10.7554/eLife.58782 (PMC8423442; doi:10.7554/eLife.58782)
Supplement: Supplementary file 1. [file elife-58782-supp1.docx]

**Supplementary File 1**: Both monkeys’ DMC task accuracy during match and non-match trials.

| monkeys |  | Monkey A |  |  | Monkey B |  |
| --- | --- | --- | --- | --- | --- | --- |
| Brain areas  Trial types | PFC | LIP | MIP | PFC | LIP | MIP |
| Match | 0.90 | 0.91 | 0.88 | 0.98 | 0.93 | 0.96 |
| Non-match | 0.97 | 0.96 | 0.98 | 0.99 | 0.99 | 0.99 |
| P value | 2.1×10^-11^ | 0.0037 | 2.2×10^-15^ | 0.045 | 0.0039 | 0.0032 |
| Sessions | 42 | 26 | 35 | 52 | 27 | 30 |
